# Discovery of Point Mutations in the Voltage-Gated Sodium Channel from African *Aedes aegypti* Populations: Potential Phylogenetic Reasons for Gene Introgression

**DOI:** 10.1371/journal.pntd.0004780

**Published:** 2016-06-15

**Authors:** Hitoshi Kawada, Yukiko Higa, Kyoko Futami, Yuto Muranami, Emiko Kawashima, Joseph H. N. Osei, Kojo Yirenkyi Sakyi, Samuel Dadzie, Dziedzom K. de Souza, Maxwell Appawu, Nobuo Ohta, Takashi Suzuki, Noboru Minakawa

**Affiliations:** 1 Department of Vector Ecology and Environment, Institute of Tropical Medicine, Nagasaki University, Nagasaki, Japan; 2 Section of Environmental Parasitology, Tokyo Medical and Dental University, Tokyo, Japan; 3 Department of Parasitology, Noguchi Memorial Institute for Medical Research, University of Ghana, Accra, Ghana; Liverpool School of Tropical Medicine, UNITED KINGDOM

## Abstract

**Background:**

Yellow fever is endemic in some countries in Africa, and *Aedes aegpyti* is one of the most important vectors implicated in the outbreak. The mapping of the nation-wide distribution and the detection of insecticide resistance of vector mosquitoes will provide the beneficial information for forecasting of dengue and yellow fever outbreaks and effective control measures.

**Methodology/Principal Findings:**

High resistance to DDT was observed in all mosquito colonies collected in Ghana. The resistance and the possible existence of resistance or tolerance to permethrin were suspected in some colonies. High frequencies of point mutations at the voltage-gated sodium channel (F1534C) and one heterozygote of the other mutation (V1016I) were detected, and this is the first detection on the African continent. The frequency of F1534C allele and the ratio of F1534C homozygotes in *Ae*. *aegypti aegypti* (Aaa) were significantly higher than those in *Ae*. *aegypti formosus* (Aaf). We could detect the two types of introns between exon 20 and 21, and the F1534C mutations were strongly linked with one type of intron, which was commonly found in South East Asian and South and Central American countries, suggesting the possibility that this mutation was introduced from other continents or convergently selected after the introgression of Aaa genes from the above area.

**Conclusions/Significance:**

The worldwide eradication programs in 1940s and 1950s might have caused high selection pressure on the mosquito populations and expanded the distribution of insecticide-resistant *Ae*. *aegypti* populations. Selection of the F1534C point mutation could be hypothesized to have taken place during this period. The selection of the resistant population of *Ae*. *aegypti* with the point mutation of F1534C, and the worldwide transportation of vector mosquitoes in accordance with human activity such as trading of used tires, might result in the widespread distribution of F1534C point mutation in tropical countries.

## Author Summary

*Aedes aegpyti* is one of the most important vectors of yellow fever and dengue fever. Pyrethroid insecticides are emerging as the predominant insecticides for vector control, and resistance of vector mosquitoes to pyrethroid is a major problem for the vector control program. Several mutations in the voltage-gated sodium channel were reported to play important roles in pyrethroid resistance of *Aedes aegypti*. Recently, a novel F1534C mutation was reported to be strongly correlated with resistance to DDT and pyrethroid. We observed a high resistance to DDT and moderate resistance to permethrin in both *Ae*. *aegypti aegypti* (Aaa) and *Ae*. *aegypti formosus* (Aaf) colonies collected in Ghana. Concurrently, high frequencies of F1534C mutations were found in the above mosquito colonies, and this was its first detection on the African continent. We found a strong linkage of F1534C mutation and the introns between exon 20 and 21 commonly found in South East Asian and South and Central American countries. The DDT and pyrethroid resistance in Ghanaian *Ae*. *aegypti* population was suggested to be caused by the introgression of Aaa genes from the above area.

## Introduction

*Aedes aegypti* (L.) is found throughout West Africa from sea-level to at least 1,220 m in Nigeria, and from the coastal swamp zone to the northern Guinea savannas. Various types of breeding sites have been reported for this species, including crab burrows, holes in trees, fallen leaves, rock pools, anthropogenic containers, etc. Transportation and urbanization of new areas are major causes of the spread of *Ae*. *aegypti* [1].

Yellow fever is endemic in Ghana and major outbreaks, which involved 319 cases and 79 deaths, occurred in 1969–1970 in the northern part of the country. In December 2011, the Ministry of Health of Ghana declared a yellow fever outbreak. Cases were recorded in three districts located in the midwestern part of the country. A total of three laboratory-confirmed cases and seven deaths were reported [2]. *Aedes aegpyti* is one of the most important yellow fever vectors implicated in the Ghana outbreaks [3]. Although there have been no reports of dengue fever outbreaks in Ghana, it has been detected in the adjacent countries of Côte d’Ivoire and Burkina Faso, both of which share borders with Ghana [4]. Increasing migration of people across the borders of these countries and the absence of organized mosquito control in Ghana might lead to dengue fever transmission in Ghana in the future [4]. A recent seroprevalence survey in Ghana revealed the presence of IgM and IgG dengue antibodies in 3.2% and 21.6% of the children, respectively, with confirmed malaria. This indicated the possible co-infection of dengue fever and malaria, and previous exposure of the children to dengue virus [5]. Although no flavivirus was detected in *Aedes* mosquitoes from the study sites, larval densities and adult biting rates of *Aedes* mosquito in study areas were thought to be sufficient to promote outbreaks of dengue fevers [4].

Pyrethroid insecticides are emerging as the predominant insecticides for vector control. Pyrethroid resistance of vector mosquitoes may become a major problem for vector control programs because there are currently no substitutes for pyrethroids [6]. Although there are some alternative chemicals to pyrethroids, no chemical seems to surpass pyrethroids in the toxicological and economical point of view. The *kdr*-type resistance has been observed in several mosquitoes, including *Anopheles gambiae* Giles [7], *Anopheles stephensi* Liston [8], *Culex quinquefasciatus* Say [9], and *Ae*. *aegypti* [10]. Several mutations in segment 6 of domain II of the voltage-gated sodium channel were reported to play important roles in pyrethroid resistance of *Ae*. *aegypti* (I1011M, I1011V, V1016G, and V1016I) [10–12]. Recently, a novel F1534C mutation in segment 6 of domain III in DDT/permethrin-resistant *Ae*. *aegypti* was reported [13,14] and this point mutation was confirmed to be strongly correlated with resistance to DDT and pyrethroid [15]. The S989P mutation in domain II, which occurs in deltamethrin-resistant *Ae*. *aegypti*, is another principal *kdr* mutation that works synergistically with the V1016G mutation [16].

The mapping of the nation-wide distribution and the detection of insecticide resistance of vector mosquitoes in Ghana will provide beneficial information for forecasting dengue and yellow fever outbreaks and developing effective control measures. Differences in the insecticide susceptibilities associated with seasonal or regional differences in the distribution of the subspecies *Ae*. *aegypti aegypti* (Aaa) and *Ae*. *aegypti formosus* (Aaf) is also of interest. Aaf which originated from African forest area is believed to be the ancestral species of *Ae*. *aegypti* s. l. Aaa is predominantly anthropophilic and adapted to the human environment, while Aaf is more associated with a forest environment [17]. Adults of Aaa prefer an indoor environments and use artificial water containers for oviposition, while Aaf prefer an outdoor environment and the forest edge and breed in natural containers such as tree holes, rock pools and plant axils. Aaa is highly susceptible to dengue and yellow fever virus, and is considered to be a more efficient virus vector than Aaf [18].

In the present paper, insecticide-susceptibility of *Ae*. *aegypti* s. l. populations (mixed populations of Aaa and Aaf) collected from used tires located in several locations in Ghana was examined. The presence of mutations in the voltage-gated sodium channel gene, S989, I1011, L1014, and V1016 and a recently identified amino acid replacement at F1534 were examined. Possible causes of insecticide resistance in Ghanaian *Ae*. *aegypti* s. l. populations were discussed based on phylogenetic analysis.

## Materials and Methods

### Ethics statement

Ethical approval for the Ghanaian field study was reviewed by Noguchi Memorial Institute for Medical Research IRB (DF22). Ethical approval for the Kenyan study was reviewed by KEMRI Ethic (CSS No. 2126).

### Collection of *Aedes aegypti* larvae from used tires

**Fig 1:**
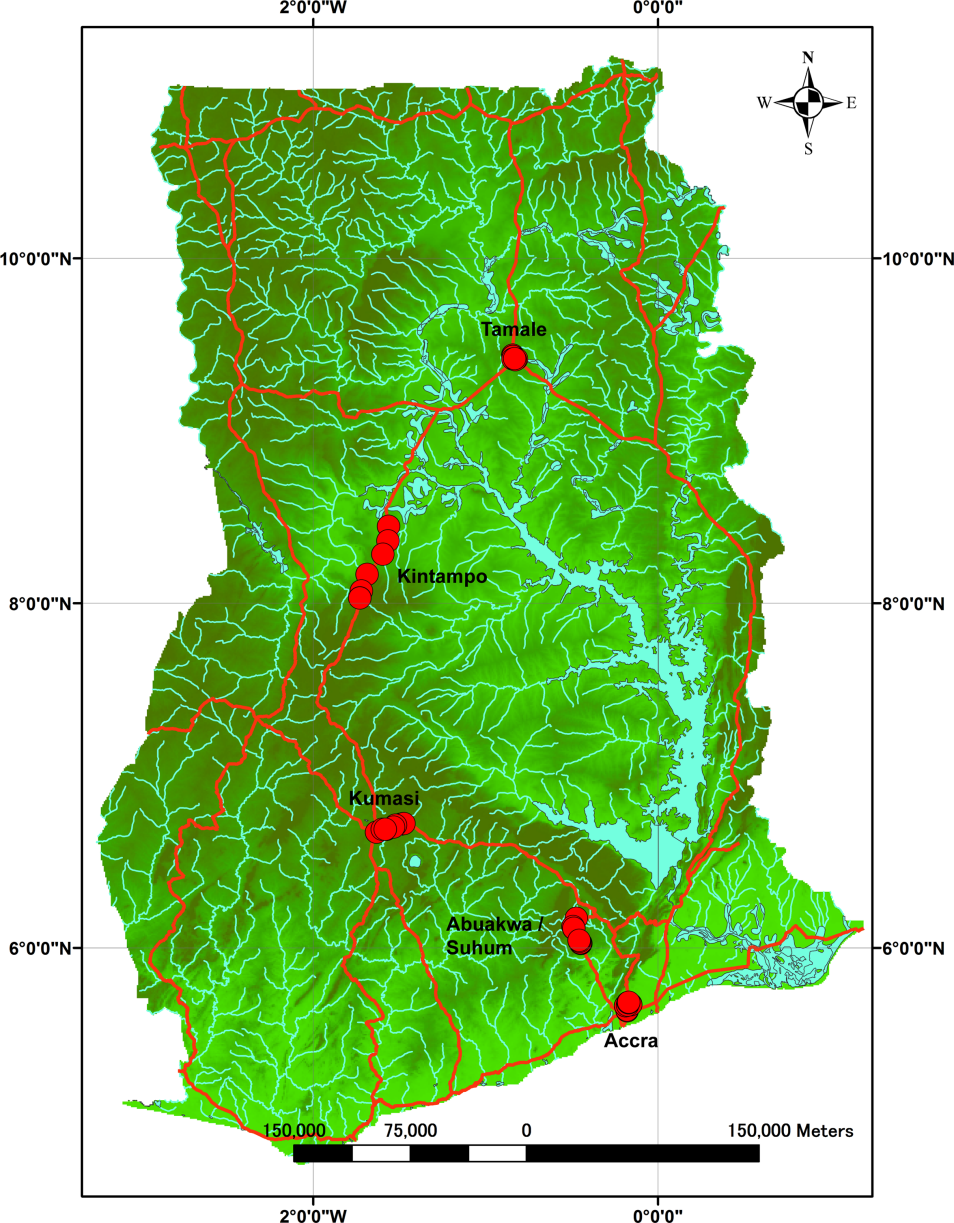
Collection sites of mosquito larvae from used tires in Ghana. The red marks indicate the collection locations. Mosquito collection points were plotted on a shape file map available from DIVA-GIS (http://www.diva-gis.org/gdata) using ArcGIS 10.2 (ESRI Japan Corp., Tokyo, Japan).

We drove along the main roads in three cities (Accra, Kumasi, and Tamale) and 2 towns (Abuakwa/Suhum and Kintampo) in Ghana, from December 4–10, 2013 (Accra, Abuakwa/Suhum, Kumasi, Kintampo, and Tamale in the beginning of the dry season) and from September 2–10, 2014 (Accra, Abuakwa, Kumasi, and Kintampo in the late rainy season) (Fig 1). Accra is the capital city located in southern Ghana and faces the Atlantic Ocean. It experiences high humidity (monthly average in 2012 and 2013, 77.4% relative humidity, Ghana Meteorological Agency, Legon-Accra, Ghana), but relatively low precipitation (monthly average 2012–2013, 46.7 mm). Kumasi, the 2nd biggest city, is located in a tropical rainforest area with high precipitation (monthly average 2012–2013, 119.6 mm) and little sunshine (monthly average 2012–2013, 4.9 hrs/day). Abuakwa/Suhum is also located in a tropical rainforest area and experiences high precipitation (annually 1270–1650 mm). Kintampo and Tamale are located in a tropical savanna climate with relatively lower humidity (monthly average 2013–2014, 60.6% for Tamale) and high temperatures (monthly average 2012–2013, 29.1°C for Tamale).

Used tires were found primarily along the periphery of repair shops, and mosquito larvae were collected from used tires with nets and dippers. We recorded the geographical location of the collection site using a global positioning system (GPS). Mosquito collection points were plotted on a shape file map available from DIVA-GIS (http://www.diva-gis.org/gdata) using ArcGIS 10.2 (ESRI Japan Corp., Tokyo, Japan). In 2013, there were 14, 11, nine, seven, and seven collection points in Accra, Abuakwa/Suhum, Kumasi, Kintampo, and Tamale, respectively. There were eight, seven, seven, and eight collection points in Accra, Abuakwa, Kumasi, and Kintampo, respectively, during 2014. Mosquito larvae collected from separate collection points in a town or city were mixed into 1 batch and reared in dechlorinated tap water at room temperature until adult emergence.

### Insecticide susceptibility tests using WHO test tubes

Tests of adult susceptibility to insecticides were performed using World Health Organization (WHO) test tube kits for the field-collected *Ae*. *aegypti* colonies. Procedures were carried out according to WHO instructions (WHO/CDS/CPC/MAL/98.12). Although the WHO recommended discriminating concentration for permethrin of 0.25% and discriminating time of contact for DDT as 30 min for *Ae*. *aegypti*, we used 1 h exposure to a higher concentration of permethrin (0.75%) and longer time (1 h) exposure for DDT (4%). Mixed adult mosquito colonies (F0) consisting of *Ae*. *aegypti aegypti* (Aaa) and *Ae*. *aegypti formosus* (Aaf) emerged from the field-collected larvae were used for the insecticide susceptibility test in the collection of 2013 (Total 138 female adults). The F1 adult mosquitoes that emerged from the eggs of F0 colonies were used for the tests in 2014 (Total 600 female adults). One- to 5-day-old unfed female mosquitoes were released into WHO test tubes and were exposed to an insecticide-impregnated paper. Basically, 3 to 4 replicates using 20 female adults per a replicate were made in the test. In the test of 2013, however, 1 to 3 replicates using the smaller number of mosquitoes were done because we could not get enough number of mosquitoes. Control tests using the papers without insecticides were done in each replication. Time to knockdown was recorded. Insects were then transferred to a clean tube and fed via cotton soaked with a 5% glucose solution, and mortality was recorded after 1 day. The time required for 50% knockdown (KT_50_) was determined, and average mortality was calculated.

### Species identification

**Fig 2:**
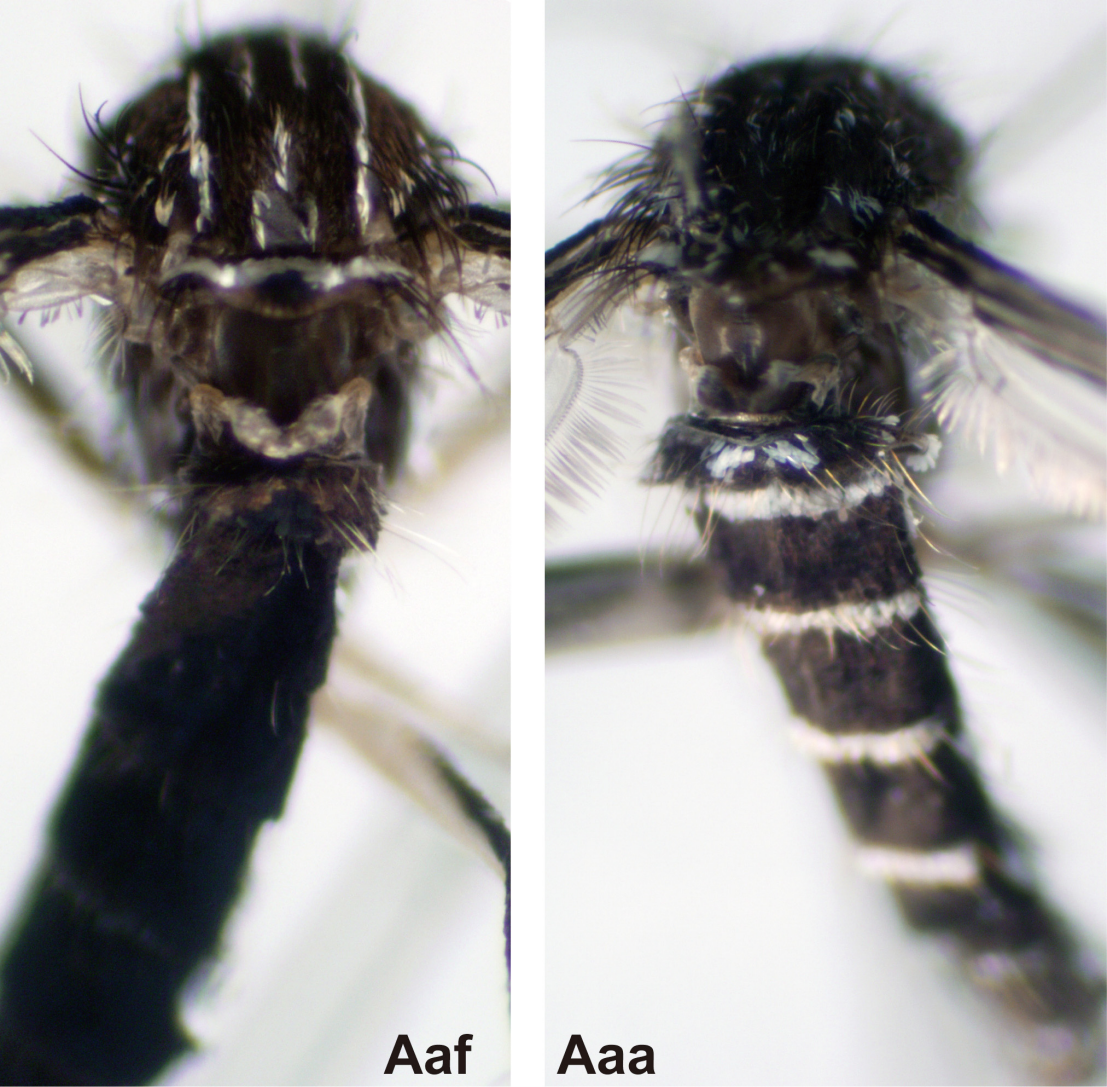
Dorsal views of abdominal tergite 1 of *Aedes aegypti aegypti* (Aaa) and *Ae*. *aegypti formosus* (Aaf).

Adult *Ae*. *aegypti* specimens were observed microscopically and identified using keys by Huang [19] into two subspecies, Aaa and Aaf. Specimens with a large, median patch of pale scales on abdominal tergite 1were identified as Aaa, and those without the median patch of pale scales were identified as Aaf (Fig 2).

### Analysis of the frequency of point mutations

Direct DNA sequencing was conducted to test for the presence of point mutations at S989, I1011, L1014, V1016, and F1534 for different adult individuals from those used in the insecticide susceptibility test. One or two legs from each specimen were placed in a 1.5-mL PCR reaction tube. The sample was homogenized in a mixture of extraction solution (20 μL) plus tissue-preparation solution (5 μL) (REDExtract-N-Amp Tissue PCR Kit; Sigma, St. Louis, MO) for extraction of DNA. The solution was heated at 95°C for 3 min and neutralized with the neutralization solution. Initial amplification was carried out using the primers AaSCF1 (AGACAATGTGGATCGCTTCC) and AaSCR4 (GGACGCAATCTGGCTTGTTA) for S989P, I1011M (or V), L1014F, and V1016G (or I); or AaSCF7 (GAGAACTCGCCGATGAACTT) and AaSCR7 (GACGACGAAATCGAACAGGT) for F1534C. The PCR mixture contained 4 μL of REDExtract-N-Amp ReadyMix (Sigma), 0.5 μM of each primer, and 1 μL of the DNA template in a total volume of 10 μL. PCR was performed under the following conditions: initial denaturation at 94°C for 3 min, 35 cycles each of 94°C for 15 s, 55°C for 30 s, and 72°C for 30 s, followed by a final elongation step at 72°C for 10 min. The amplified fragments of the expected size were purified with ExoSAP-IT (USB Corporation, Cleveland, OH) at 37°C for 30 min, and then 80°C for 15 min. DNA sequencing was carried out using the primers AaSCF3 (GTGGAACTTCACCGACTTCA) and AaSCR6 (CGACTTGATCCAGTTGGAGA) for S989P, I1011M (or V), L1014F; and V1016G (or I), or AaSCR8 (TAGCTTTCAGCGGCTTCTTC) for F1534C. A BigDye Terminator v 3.1 Cycle Sequencing Kit (Applied Biosystems Japan Ltd., Tokyo, Japan) was used for DNA sequencing, according to the manufacturer’s instructions. Two micromoles of each primer were added to a tube, making total mixture volume 10 μL. PCR was performed under the following conditions: initial denaturation at 96°C for 1 min. 25 cycles each of 96°C for 10 s, 50°C for 5 s, and 60°C for 2 min. Direct DNA sequencing was performed on the 3730 DNA Analyzer (Applied Biosystems Japan Ltd.). The electropherogram of the targeted amino acid replacement was analyzed with MEGA 6.0 public domain software (http://www.megasoftware.net/). The unique DNA haplotype sequences were deposited in GenBank.

### Phylogenetic analysis

The genetic diversities in the introns between 1015V and 1016V (the sequences produced by the direct sequence described as above) located in the domain II area of the voltage-gated sodium channel in the field-collected *Ae*. *aegypti* specimens from Africa, Asia, and South and Central America, along with other genetic information for this species in GenBank were analyzed to determine the genetic affinity of Ghanaian *Ae*. *aegypti* populations (423 Aaa and 336 Aaf) to other populations. Newly determined sequences (Ghana, Malawi, Zambia, Zimbabwe, Kenya, Philippines, Singapore, Vietnam, and El Salvador) and the sequences obtained from GenBank (Brazil, India, Indonesia, and Myanmar) (S1 Table) were aligned initially using MEGA version 6 [20], and subsequently modified manually if needed. The alignment was performed for a 263 bp of fragment with gaps (total fragment lengths were from 228 to 250 bp). Thus, two datasets were prepared. In the first dataset, all indels were completely removed from the fragment (final length was 207 bp). In the second, those gaps were treated as a 5th variable in maximum parsimony analysis. A single gap was assumed to have evolved once, whereas a longer indel was assumed to have been caused by either one- or two-time events by comparing same sites of other sequences.

### Statistical analysis

KT_50_ (time to cause 50% knockdown) was calculated using the Bliss' probit method [21]. Chi-square tests were used for the comparison of subspecies composition of Aaa and Aaf between the collection in 2013 and 2014, and the comparison of insecticide susceptibility between the two subspecies. For the two phylogenetic datasets, a total of four phylogenetic analyses were conducted: 1) maximum parsimony analysis (MP), 2) maximum likelihood analysis (ML), and 3) neighbor joining analysis (NJ) were all conducted using the 1st data, and 4) MP was also conducted using the 2nd data. Based on the model selection program of MEGA, the Tamura 3-parameter model was the evolution model used. The first three analyses were conducted by MEGA, whereas the MP tree for the 2nd dataset was constructed using PHYLIP3.69 (http://evolution.genetics.washington.edu/phylip.html). For the all constructed trees, Bootstrap replication was operated for 1,000 times to calculate how strongly the branches were supported.

## Results

### Subspecies composition

**Fig 3:**
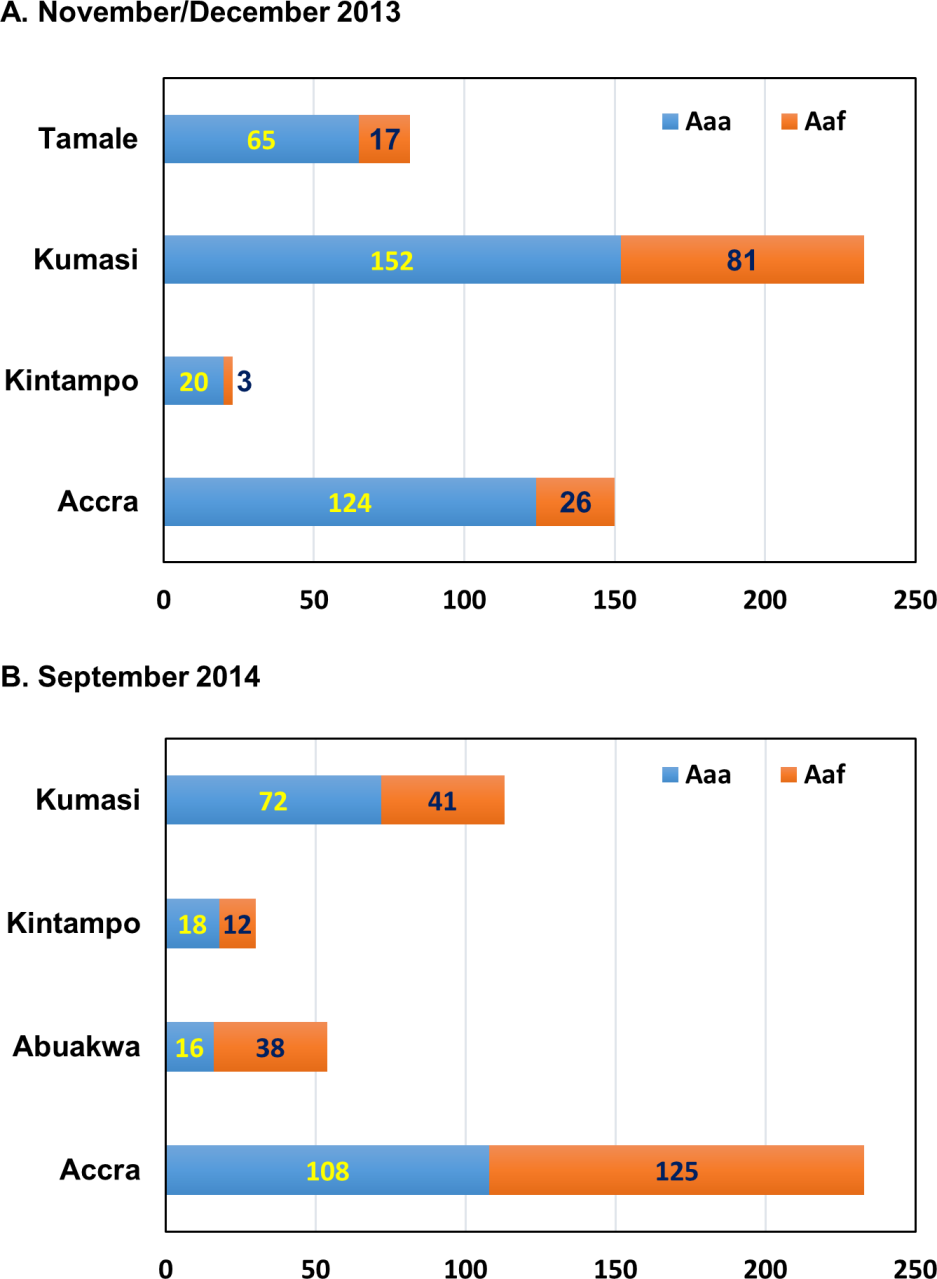
Species composition of *Ae*. *aegypti aegypti* (Aaa) and *Ae*. *aegypti formosus* (Aaf) collected from used tires in Ghana. Larval collection from used tires was performed in November and December, 2013 (A) and September, 2014 (B).

Subspecies composition of Aaa and Aaf based on the identification criteria by Huang [20] is shown in Fig 3. In the first collection in November and December 2013, Aaa appeared dominant in Accra (82.7%), Kintampo (87.0%), and Tamale (79.3%), whereas the composition rate was relatively lower in Kumasi (65.2%). Conversely, in the 2nd collection performed in September 2014, the composition rates of Aaa in Accra (46.4%), Abuakwa (25.5%), and Kintampo (60.0%) were lower than those in 2013 collection, while the composition rate in Kumasi was in the same range as in 2013 (63.7%). The composition rates of Aaa were significantly lower in Accra (χ^2^ = 16.8, df = 1, *P* < 0.0001) and Kintampo (χ^2^ = 4.66, df = 1, *P* = 0.031) as compared to those in 2013, whereas no such significant change in composition was observed in Kumasi (χ^2^ = 0.077, df = 1, *P* = 0.78).

### Insecticide susceptibility of Ghanaian *Ae*. *aegypti* colonies

**Fig 4:**
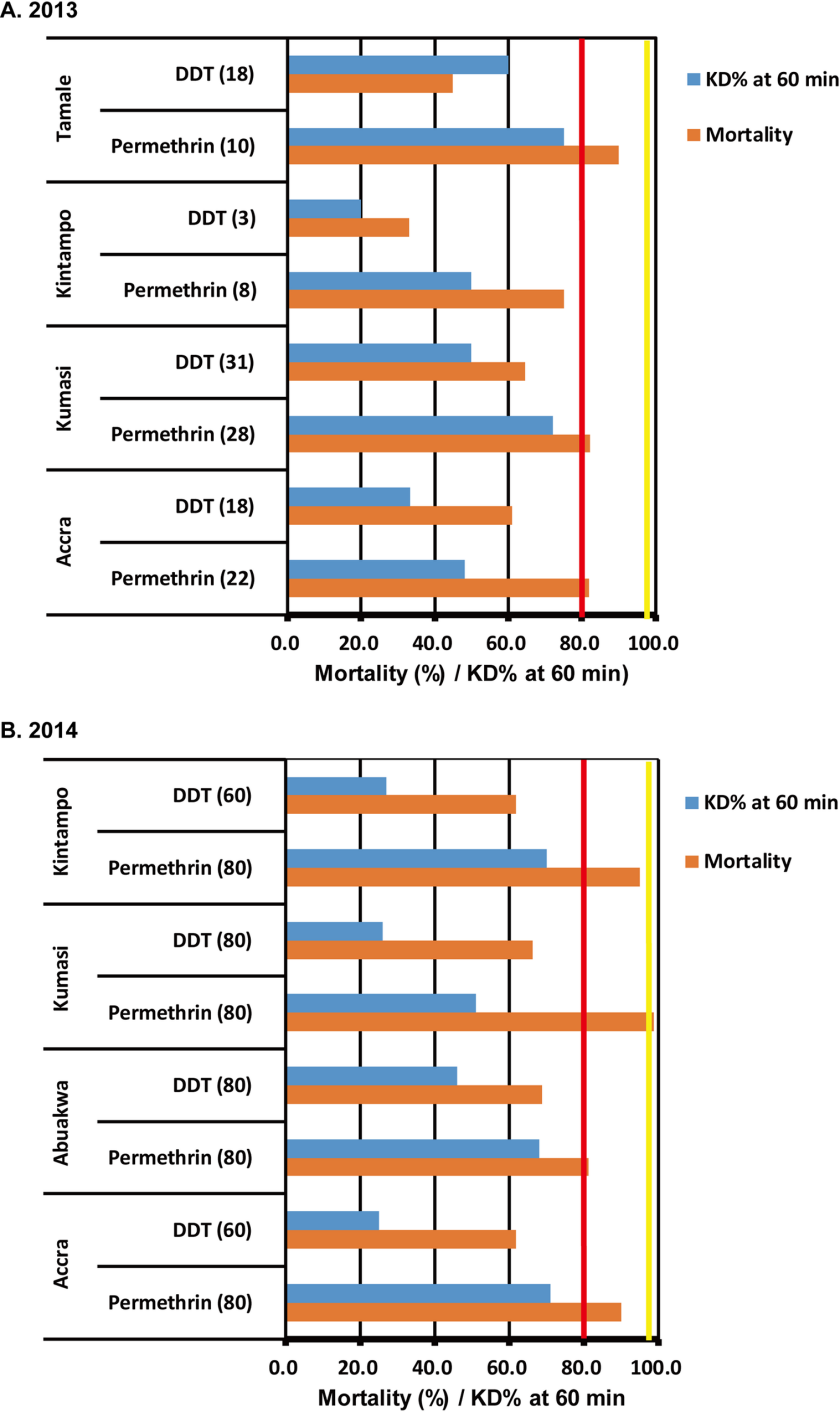
Insecticide susceptibility of adult *Ae*. *aegypti* (mixed colonies of *Ae*. *aegypti aegypti* and *Ae*. *aegypti formosus*) by the WHO tube test. Figures in parenthesis indicate the number of mosquitoes used for the test. Mosquito colonies mixed with *Ae*. *aegypti aegypti* and *Ae*. *aegypti formosus* emerged from the field collected larvae were used for the test in the collection in 2013 (Fig 4A). The F1 adult mosquitoes emerged from the eggs collected from field collected larvae were used for the test in the collection in 2014 (Fig 4B). A mortality rate between 98% (yellow line) and 100% is considered to indicate susceptibility; 80 (red line)– 97% mortality suggests the possibility of resistance that needs to be confirmed. Mortality <80% indicates resistance by WHO criteria.

Totally, there was no difference in the susceptibility to permethrin (χ^2^ = 0.010, df = 1, *P* = 0.92 in 2013 collection; χ^2^ = 1.46, df = 1, *P* = 0.23 in 2014 collection) and DDT (χ^2^ = 1.26, df = 1, *P* = 0.26 in 2013 collection; χ^2^ = 0.021, df = 1, *P* = 0.88 in 2014 collection) between Aaa and Aaf used for the susceptibility test. The susceptibilities to the insecticides were, therefore, compared with mixed colonies of both subspecies (Fig 4). High resistance to DDT (less than 70% mortality at 1 h contact) was observed in all mosquito colonies. KT_50_s with DDT for these colonies were >60 min, except for Tamale (2013) and Kumasi (2013) colonies (KT_50_ was 56.4 and 58.2 min, respectively), indicating low knockdown ability of DDT against these colonies, as well as low killing rates. Susceptibilities to permethrin (0.75%) were relatively higher in all colonies as compared to those for DDT. Resistance to permethrin was, however, suspected in the Accra (<90% mortality) and Abuakwa colony (81.3% mortality). For the Kumasi and Kintampo colonies collected in 2014, mortalities with permethrin were higher than the other colonies, but were less than 100% (98.8% and 95.0% mortality, respectively), indicating the possible existence of resistance or tolerance to permethrin.

### Point mutations in the voltage-gated sodium channel found in Ghanaian *Ae*. *aegypti*

Total 759 specimens (262 in 2013 collection and 497 in 2014 collection) were sequenced. No mutation at S989, I1011, or L1014 was detected among 707, 756, and 734 mosquitoes sequenced, respectively. Conversely, F1534C mutations were detected at high frequency: 294 homozygous and 259 heterozygous mutations among 759 mosquitoes sequenced (accession No. LC050217, LC050218). Table 1 shows the homozygous percentages and allelic frequencies of point mutations at 1534F in Aaa mosquitoes collected from five different places in Ghana. Allelic frequencies of F1534C mutations were higher in Accra (68.4%), Kumasi (64.6%), and Kintampo (58.3%) than other places. The allelic frequencies of F1534C mutations in Aaf were also higher in Accra (52.6%), Kumasi (60.0%), and Kintampo (45.2%) than other places (Table 2). The frequency of F1534C allele and the ratio of F1534C homozygous mosquitoes in Aaa was significantly higher than that in Aaf in the mixed populations from all collection places in 2013 and 2014 (Table 3). Additionally, one heterozygote point mutation (V1016I, accession No. LC050223) was found in Accra among 732 mosquitoes (Table 4). Homozygous F1534C was concurrently found in this individual (Aaa). No V1016I mutation was found in Aaf (Table 5).

**Table 1:** Number of genotypes, and homozygous and allelic percentage of the point mutations (F1534C) in the voltage-gated sodium channel of *Aedes aegypti aegypti* collected in Ghana.

Collection Place	Total	1534F			Homozygous %	Allelic %
		F1534C / F1534C	F1534C / +	+ / +		
Accra	171	97	40	34	56.7	68.4
Abuakwa	6	1	2	3	16.7	33.3
Kumasi	158	67	70	21	42.4	64.6
Kintampo	24	14	0	10	58.3	58.3
Tamale	64	10	31	23	15.6	39.8

**Table 2:** Number of genotypes, and homozygous and allelic percentage of the point mutations (F1534C) in the voltage-gated sodium channel of *Aedes aegypti formosus* collected in Ghana.

Collection Place	Total	1534F			Homozygous %	Allelic %
		F1534C / F1534C	F1534C / +	+ / +		
Accra	151	51	57	43	33.8	52.6
Abuakwa	33	0	6	27	0.0	9.1
Kumasi	115	48	42	25	41.7	60.0
Kintampo	21	6	7	8	28.6	45.2
Tamale	16	0	4	12	0.0	12.5

**Table 3:** Chi-square analysis of the number of F1534C allele and F1534C homozygotes between *Aedes aegypti aegypti* (Aaa) and *Ae*. *aegypti formosus* (Aaf) collected in Ghana (2013–2014).

Subspecies	No. of Allele			No. of Homozygotes		
	F1534C	+	χ^2^, df, *P*	F1534C/F1534C	F1534C/+ or +/+	χ^2^, df, *P*
Aaa	521	325	25.9, 1, < 0.001	189	234	14.2, 1, < 0.001
Aaf	326	346		105	231	

**Table 4:** Number of genotypes, and homozygous and allelic percentage of the point mutations (V1016I) in the voltage-gated sodium channel of *Aedes aegypti aegypti* collected in Ghana.

Collection Place	Total	1016V			Homozygous %	Allelic %
		V1016I / V1016I	V1016I / +	+ / +		
Accra	160	0	1	159	0.0	0.3
Abuakwa	4	0	0	4	0.0	0.0
Kumasi	72	0	0	72	0.0	0.0
Kintampo	18	0	0	18	0.0	0.0
Tamale	62	0	0	62	0.0	0.0

**Table 5:** Number of genotypes, and homozygous and allelic percentage of the point mutations (V1016I) in the voltage-gated sodium channel of *Aedes aegypti formosus* collected in Ghana.

Collection Place	Total	1016V			Homozygous %	Allelic %
		V1016I / V1016I	V1016I / +	+ / +		
Accra	159	0	0	159	0.0	0.0
Abuakwa	33	0	0	33	0.0	0.0
Kumasi	176	0	0	176	0.0	0.0
Kintampo	32	0	0	32	0.0	0.0
Tamale	16	0	0	16	0.0	0.0

### Phylogenetic analysis of Ghanaian *Ae*. *aegypti*

**Fig 5:**
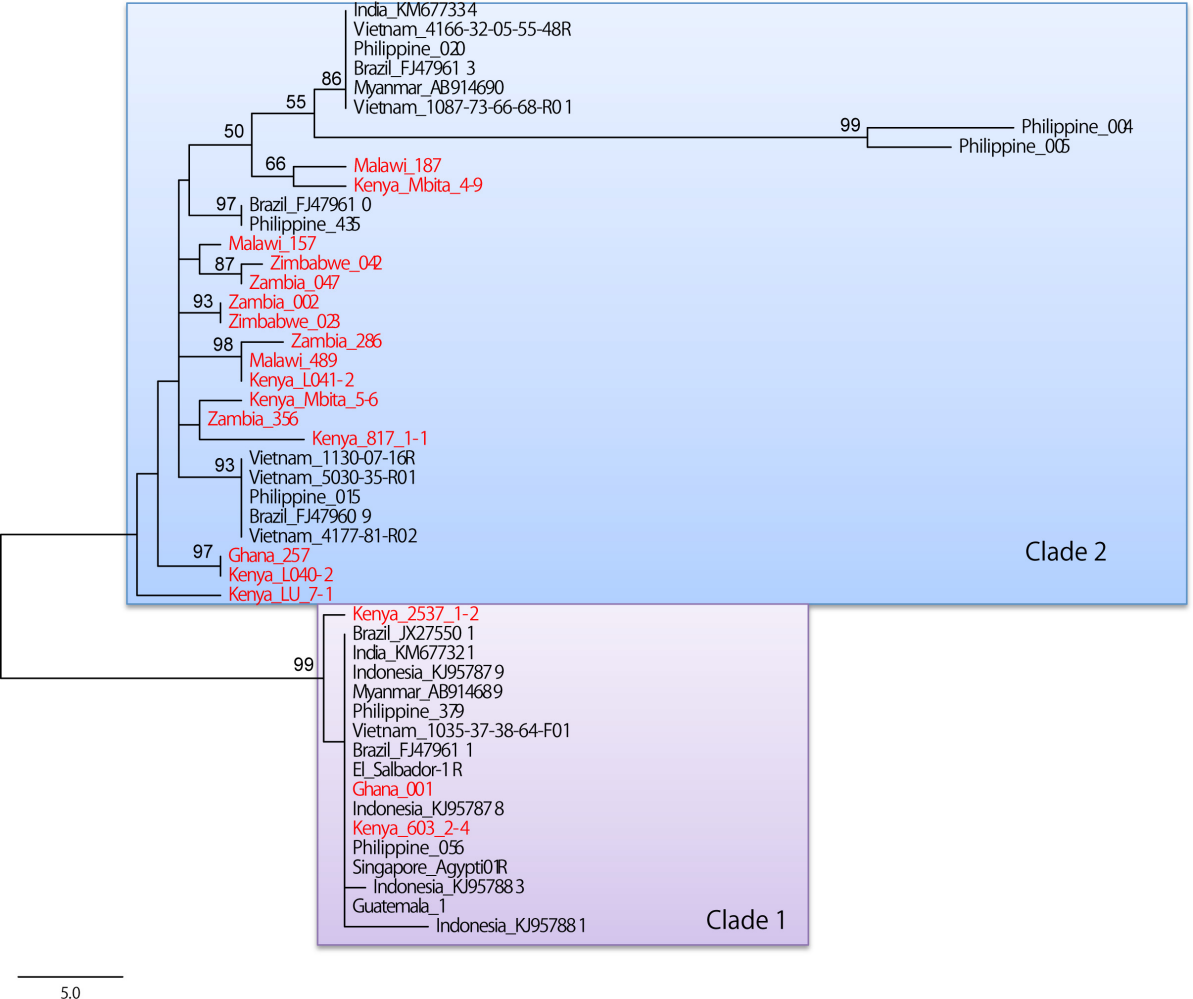
Maximum Parsimony analysis of *Ae*. *aegypti*. The evolutionary history was inferred using the Maximum Parsimony method using MEGA6. Tree No. 1 out of the four most parsimonious trees (length = 116) is shown. The percentages of replicate trees in which the associated sequences clustered together in the bootstrap test (1,000 replicates) are shown next to the branches [56]. The MP tree was obtained using the Subtree-Pruning-Regrafting (SPR) algorithm (pg. 126 in the manual of PHYLIP version 3.69) with search level 1 in which the initial trees were obtained by the random addition of sequences (10 replicates). The tree is drawn to scale, with branch lengths calculated using the average pathway method (see pg. 132 in the manual of PHYLIP version 3.69) and are in the units of the number of changes over the whole sequence. The analysis involved 48 nucleotide sequences. All positions containing gaps and missing data were eliminated. There were a total of 209 positions in the final dataset.

We detected two types of introns between exon 20 and 21 in the Ghanaian *Ae*. *aegypti* populations (183 specimens of Aaa and Aaf): Ghana 001 (250 bp, Accession No. LC036551) and Ghana 257 (234 bp, Accession No. LC036552). The point mutations at 1534F (F1534C) on exon 31 were found to be strongly linked with the intron of the former group (Group A in Table 6). When the two types of the intron were treated as two alleles, strong linkage disequilibrium was observed between mutation at 1534F and the two types of intron (using Genepop, G-test with 100 repeats of 10000 iteration per batch, P < 0.001). All phylogenetic trees showed similar topology (Fig 5 and S1–S5 Figs). Thus, we show one of the MP trees constructed using the 1st dataset (no indel) in Fig 5. The sequences from each geographic area were not in the same clade, and were distributed paraphyletically. Two large clades were observed: Clade 1 consisted of the sequences from southeastern Asia and the South and Central America with two from Kenya and one from Ghana, and Clade 2 consisted of the remaining African samples and strongly supported Asian or American branches. Ghana 001 (Group A in Table 6) shared the same sequence with most of other Asian and South-American sequences within Clade 1. These 2 clades were strongly supported by a consensus tree of four parsimonious trees using the 1st dataset (S2 Fig). Most African sequences were placed in Clade 2, and Asian and American sequences were distributed in three strongly supported monophyletic clades within Clade 2. When the consensus tree of MP analysis using the 1st dataset was compared to the consensus tree using the 2nd dataset (indel was treated as 5th variable), only the branch position of clade 1 was changed (S1 and S5 Figs). ML and NJ trees also showed similar topology, with a clade consisting of two Kenyan and one Ghanaian sequences and another clade consisting of the remaining sequences (S3 and S4 Figs).

**Table 6:** Chi-square analysis of the linkage of F1534C mutation with the 2 types of intron between exon 20 and 21 in Ae. aegypti^1)^ collected in Ghana.

Point mutation at 1534F	Type of the intron between exon 20 and 21			χ^2^, df, *P*
	Group A^2)^	Group B^2)^	Group A/Group B^3)^	
F1534C / F1534C	110	0	6	
F1534C / +	1	0	42	161.14< 0.01
+ / +	1	2	21	

^1)^ Mixed specimens of 97 Aaa and 86 Aaf were used for the analysis.

^2)^ According to the classification by Martins et al. [49]; introns belong to group A (Ghana 001) and B (Ghana 257) have length of 250 bp and 234 bp, respectively.

^3)^ Heterozygotes.

## Discussion

The rainy season in Ghana starts in March and lasts until the end of October. The collection period in our survey was November and December 2013 and September 2014. Therefore, our collection dates corresponded to the beginning of the dry season and the late rainy season, respectively. Accordingly, the proportion of the *Ae*. *aegypti aegypti* (Aaa) collected in samples from used tires in Accra and Kintampo were higher in the dry than rainy season, although no such difference was observed in Kumasi (Fig 3). The same seasonal shift in subspecies abundance was reported for *Ae*. *aegypti* s. l. in tree holes and fruit husks in southeastern Senegal where most of the *Ae*. *aegypti* s. l. in the wet season were subspecies *formosus* [22]. It is interesting that there was no such seasonal difference in subspecies composition in Kumasi. This appeared attributable to the relatively consistent precipitation in Kumasi throughout the year (monthly average precipitation in 2012 and 2013 was 162.4 mm in the rainy season and 88.9 mm in the dry season; Ghana Meteorological Agency, Legon-Accra, Ghana) as compared to Accra (68.2 mm and 29.6 mm for the wet and dry season, respectively) and Kintampo (75.1 mm and 46.6 mm for the wet and dry season, respectively, in adjacent Tamale).

Trpis and Hausermann collected larvae of *Ae*. *aegypti* s. l. in three principal habitats (domestic, peridomestic, and feral) in the Rabai area in eastern Kenya [23]. The mosquitoes from the domestic habitat were represented by the domestic form, Aaa, and the feral mosquitoes from tree holes were represented by the feral subspecies, Aaf. A hybridization experiment showed that house-entering behavior was genetic, and the percentage entering houses was highest in the domestic Aaa populations and lowest in the feral Aaf populations. The authors suggested that the populations from the peridomestic habitat may represent hybrids between the domestic and feral forms. The involvement of a gene expression related the odorant receptor of human-specific odor component (sulcatone) was also suggested to explain the behavioral difference between the two subspecies [24]. Sylla et al. found that both Aaa and Aaf may survive the tropical dry season in natural habitats, such as tree holes and husks, in West Africa [22]. Our finding, that both of the subspecies were found in an artificial habitat (used tires) provides another contrasting trend to that of previous studies in East Africa that reported household containers were the exclusive larval habitat for Aaa and tree holes the predominant habitat for Aaf [17, 23].

Source reduction and use of insecticides, such as organophosphates, carbamates, and pyrethroids, were recommended by the WHO as preventive control measures for the vector mosquitoes of yellow fever. Use of organochlorine compounds, however, is not recommended because of widespread resistance of *Ae*. *aegypti* to these compounds in the 1980s [25]. Thermal fogging, mist blowers, Ultra low volume (ULV) spray, and indoor residual spraying (IRS) with the above insecticides (organophosphates, carbamates, and pyrethroids) were recommended by the WHO as emergency control measures for *Ae*. *aegypti*. Resistance of *Ae*. *aegypti* to hexachlorocyclohexane (HCH) in Navrongo, Kassena and dieldrin resistance in upper region of Lawra were reported in Ghana in 1971 [25]. The present study is perhaps the first to report the resistance of *Ae*. *aegypti* s. l. to DDT and permethrin in Ghana, although DDT resistance in *Ae*. *aegypti* was common in countries adjacent to Ghana, such as Côte d'Ivoire (1968), Togo (1969), and Benin (1968) [25]. High resistance to DDT seems to be widespread and resistance to pyrethroids is also suspected to be common in Ghana. Although the concentration of DDT and pyrethroids used and how they were applied for the control of *Ae*. *aegypti* in Ghana is unknown, it is clear that these insecticides were one of the causative factors in the resistance of *Ae*. *aegypti* s. l. populations in Ghana. Organochlorine pesticides were most popular and extensively used by farmers in Ghana with lindane commonly used for pest control on cocoa, vegetables, and maize, and endosulfan on cotton, vegetables, and coffee. DDT and lindane were once employed to control ectoparasites of farm animals and pets in Ghana [26]. Lambda-cyhalothrin and cypermethrin are used by vegetable growers on tomato, pepper, okra, eggplant, cabbage, and lettuce farms [26]. The contamination caused by the aforementioned pesticides to the breeding area might have served as selection pressure for the development of the resistance in *Ae*. *aegypti* populations.

Additionally, indirect effects of long lasting insecticidal nets (LLINs), IRS, and other insecticide treatment for malaria control have contributed to the development of DDT and pyrethroid resistance as previously reported in *Ae*. *aegypti* populations in Vietnam [27–30]. The malaria vector *Anopheles gambiae* s. l. in southwestern Ghana has developed a high resistance to DDT and pyrethroid insecticides in an area where the species was susceptible to these chemicals just a decade ago [31, 32]. The use of insecticides, such as LLINs and IRS, in the Ghana National Malaria Control Program is believed to be the major cause for the cross-resistance between DDT and pyrethroids. This was mainly attributable to the *kdr* gene [31] as reported in the adjacent countries of Mali [33] and Burkina Faso [34]. Pyrethroid treatment for malaria vector control appears to have been intensively conducted in the interior and along the periphery of human habitation areas, where the breeding and resting sites of *Ae*. *aegypti* are located. This likely contributed to the strong selection pressure toward *Ae*. *aegypti* (especially Aaa) because this species is domestic and endophagic. Extensive use of DDT for malaria control before it was banned may have also contributed to the development of pyrethroid resistance in *Ae*. *aegypti* because the target site (i.e., the voltage-gated sodium channel) is common to both DDT and pyrethroids.

F1534C mutations were reported worldwide (i.e., South Asian, South East Asian, South and Central American countries and Macaronesian islands). After the first description of the F1534C point mutation in *Ae*. *aegypti* collected in Thailand [13, 14], the same mutation was reported in succession in Vietnam [27], Grand Cayman Island [15], Madeira Island [35], Brazil [36], Myanmar [37], Venezuela [38], India [39], and Malaysia [40]. The mutations at 1016V were also reported worldwide. Two different types of the mutation at the same locus are distributed independently. Valine to glycine replacements (V1016G) are commonly distributed in South East Asia [37, 40–43], whereas valine to isoleucine replacements (V1016I) are common in South and Central America [11, 36, 38, 44–46].

The present study provides the first description of F1534C and V1016I mutations found in African *Ae*. *aegypti* s. l. populations. Accra and Kumasi are the two largest cities in Ghana, each home to one to two million people. Tamale is the 3rd largest city with a population of approximately 400,000 people. Kintampo and Abuakwa, both of which have populations of approximately 40,000, are much less populated compared to the other sampled cities. Allelic frequencies of F1534C and percentage of homozygous individuals of the same point mutation were higher in the two large cities, Accra and Kumasi, than the other collection locations. Interestingly, both the allelic frequency and homozygous percentage of F1534C in Aaa was significantly higher than that in Aaf, though we could not observe the significant difference in the susceptibility to DDT and permethrin between the two subspecies. Above discrepancy might suggest that the F1534C mutation is not a single resistance mechanism but is combined with the other unknown mechanisms such as metabolic factors etc. Recently, some reports called attention to the role of glutathione-S-transferases (GST) in the cross resistance between DDT and pyrethroids in mosquitoes [47, 48]. Riveron et al. demonstrated that the single amino acid change in GST gene (L119F) confers high level of metabolic resistance to DDT in *Anopheles funestus* [47]. The authors also showed that this mutation strongly related to the metabolism of permethrin. Several Epsilon GST genes were reported to play a role in pyrethroid resistance in *Ae*. *aegypti* [48]. The above new findings, as well as the other metabolic factors, should be taken into consideration for further study.

Martins et al. reported two types of haplotype group A (250 pb) and B (234 pb) in the intron between exons 20 and 21 on domain II of the voltage-gated sodium channel with pronounced differences in both sequence and size in Brazilian *Ae*. *aegypti* [49]. The introns in the sequence of accession No. FJ479611 referred in S1 Table and Fig 5 correspond to the haplotype group A and those of FJ479609, FJ479610, and FJ479613 correspond to the haplotype group B. The authors also noted point mutations at 1011I (I1011M) on exon 20 appeared in half of the group A sequences, whereas no such mutation occurred in group B sequences. The same kind of the evidence of linkage equilibrium was reported by Saavedra-Rodriguez et al. The authors found the same intron as reported above (group A) strongly linked with V1016I mutation and hypothesized that a genetic sweep of the V1016I allele and its proximate intron sequences has occurred through DDT and subsequent pyrethroid selection [11]. In the present study, we could detect the same two types of intron in the Ghanaian *Ae*. *aegypti* populations: Ghana 001 for group A and Ghana 257 for group B. Interestingly, the point mutations at 1534F (F1534C) on exon 31 were found to be strongly linked with the intron of group A. Furthermore, phylogenetic analysis using this intron in the present study clearly showed that the two Ghanaian haplotypes belonged to two haplotype groups (Clades 1 and 2). Given *Ae*. *aegypti* was originally from Africa, Clade 2 is thought to be the ancestral clade of Clade 1 because Clade 2 contained most African haplotypes. Interestingly, one of the two Ghanaian haplotypes (Ghana 001) and two Kenyan haplotypes were placed in Clade 1, apparently suggesting those haplotypes were introduced from other continents, such as Asia or South or Central America.

*Aedes aegypti* is thought to have originated on the African continent. The sub-Saharan part of the continent still contains Aaf, which is believed to be the ancestral species of *Ae*. *aegypti* s. l. The subspecies that has been domesticated and adapted to anthropomorphic environments (Aaa) expanded its habitat around human domiciles and has been dispersed by human movement. *Aedes aegypti aegypti* spread to the western hemisphere in the 17th centuries, to the Mediterranean coastal area in the 18th centuries, and to tropical Asian and Pacific islands in the 19th to 20th centuries. This subspecies was eradicated from the Mediterranean area in 1950s and from south America from 1950 to 1960. However, it has reinfested most of the countries from which it was eradicated [50]. The *Ae*. *aegypti* eradication program, initiated by the Pan American Health Organization (PAHO) in the 1940s and 1950s to prevent urban epidemics of yellow fever, was successful in most of the countries in South and Central America, resulting in a dramatic decrease in the distribution of mosquito populations. However, the discontinuation of the eradication program in 1970s led the reinfestation of the mosquitoes and *Ae*. *aegypti* regained a similar distribution to that of the 1940s by 1995 [51]. The worldwide eradication programs, presumably with organochlorine insecticides, in 1940s and 1950s might have caused high selection pressure on the mosquito populations and expanded the distribution of insecticide-resistant *Ae*. *aegypti* populations [25]. Selection of the F1534C point mutation could be hypothesized to have taken place during this period. DDT resistance in *Ae*. *aegypti* was first reported in the Caribbean countries in the 1950s and the resistance persists in almost all regions that had achieved *Ae*. *aegypti* eradication, despite the fact that DDT is no longer used. DDT resistance, as well as the F1534C point mutation might have been maintained in the *Ae*. *aegypti* populations by selection pressures from pyrethroid insecticides, such as permethrin, as both insecticides have the same target site [15].

Used and discarded tires are one of the most important breeding sites for *Ae*. *aegypti*. They provide a habitat for the larvae and are capable of supporting larval development immediately after they are discarded. Accumulation of microorganisms in time improves the breeding environment [52]. It is noteworthy that there has been worldwide focus on the dispersal of containers breeding mosquitoes in the used tires for the past three decades [53]. The mosquito species that has played the leading part in the above event are *Ae*. *albopictus* (Skuse), which together with the other four Japanese mosquito species was thought to have arrived at the western coastal ports of the United States in used tires by 1983 and were widely distributed in the United States and Brazil by 1986 [54]. Since 1986, tire shipments infested with *Ae*. *albopictus* have been found in the South and Central America, South and West Africa, Oceania, and European countries [54]. Used tire trading is worldwide with complicated commercial networks, including those in African countries, such as South Africa, Kenya, Uganda, Niger, Nigeria, and Ghana [53, 54]. The history of the worldwide invasion by *Ae*. *aegypti* in association with human activity might be longer than that of *Ae*. *albopictus*.

The worldwide transportation of vector mosquitoes in accordance with human activity such as trading of used tires, might result in the introgression of Asian or Central or South American type haplotype into Ghanaian *Ae*. *aegypti* population. It is not known whether Ghanaian F1534C mutations "hitched a ride" with the above haplotypes or they were selected convergently after the above introgression. However, strong linkage disequilibrium between F1534C mutation and intron haplotypes may support introgression of the mutation. Phylogeographic analyses with other loci (e.g. mtDNA, ITS) and detailed population genetic analyses on intron (e.g. mismatch analysis) could provide more evidence supporting the hypothesis. Discovery of the V1016I mutation, although it was only 1 heterozygote, and co-occurrence of this mutation with F1534C might be noteworthy. Recently, Linss et al. detected the widespread co-occurrence of V1016I and F1534C point mutations in *Ae*. *aegypti* populations in Brazil [36]. The same co-occurrence of the two point mutations were reported in Grand Cayman Island [15]. The high frequency of F1534C and the co-occurrence of V1016I with this mutation, therefore, might explain one of the possible history of introduction of these mutations into the Ghanaian *Ae*. *aegypti* population from South and Central America. The importance of the sequential evolution of F1534C and V1016I was advocated in Mexican *Ae*. *aegypti* population [55]. V1016I mutation was unlikely to have evolved independently because of low fitness, while F1534C mutation evolved first but conferred only a low level resistance. V1016I mutation then rapidly evolved from 1016V/F1534C haplotype under the high pressure of pyrethroids since these double mutants confer higher pyrethroid resistance. The authors suggested that knowledge of the frequencies of mutations in both S6 in domains II and III are important to predict the potential of a population to evolve *kdr*. They also sounded an alarm that susceptible populations with high 1016V/F1534C frequencies are at high risk for *kdr* evolution [55]. We, therefore, have to pay great attention to the genomic status in Ghanaian *Ae*. *aegypti* populations for predicting the evolution of pyrethroid resistance.

## Supporting Information

S1 Table Information for the sequences between exon 20 and 21 of *Ae*. *aegypti* used for phylogenetic analysis. (DOC)

S1 Fig Maximum Parsimony analysis of *Ae*. *aegypti* (no indel). Other trees inferred using the Maximum Parsimony method are shown. Because of differences in two of them, we show only two trees. A) MP tree in which Philippine clade was placed at the base of clade 2, B) MP tree in which Philippine clade was NOT placed at the base of clade 2. The percentage of replicate trees in which the associated sequences clustered together in the bootstrap test (1,000 replicates) are shown next to the branches [56].
(TIF)

S2 Fig Consensus tree of Maximum Parsimony analysis of *Ae*. *aegypti* (no indel). Evolutionary history was inferred using the Maximum Parsimony method. The consensus tree inferred from the four most parsimonious trees constructed using MEGA6 is shown. Branches corresponding to partitions reproduced in less than 50% trees (reproduced in only one tree) are collapsed. The percentages of parsimonious trees in which the associated sequences clustered together are shown next to the branches.
(TIF)

S3 Fig Neighbor-Joining tree of intron of *Ae*. *aegypti* (no indel). Evolutionary history was inferred using the Neighbor-Joining method [57]. The optimal tree with the sum of branch length = 0.566 is shown. The percentages of replicate trees in which the associated sequences clustered together in the bootstrap test (1,000 replicates) are shown next to the branches [54]. The tree is drawn to scale, with branch lengths in the same units as those of the evolutionary distances used to infer the phylogenetic tree. The evolutionary distances were computed using the Tamura 3-parameter method [58] and are in the units of the number of base substitutions per site. The analysis involved 48 nucleotide sequences. All positions containing gaps and missing data were eliminated. There were a total of 209 positions in the final dataset. Evolutionary analyses were conducted in MEGA6.
(TIF)

S4 Fig Maximum Likelihood tree of intron of *Ae*. *aegypti* (no indel). Evolutionary history was inferred by using the Maximum Likelihood method based on the Tamura 3-parameter model [58]. The tree with the highest log likelihood (-899.5569) is shown. The percentages of replicate trees in which the associated sequences clustered together in the bootstrap test (1,000 replicates) are shown next to the branches [56]. Initial tree(s) for the heuristic search were obtained by applying the Neighbor-Joining method to a matrix of pairwise distances estimated using the Maximum Composite Likelihood (MCL) approach. The tree is drawn to scale, with branch lengths measured in the number of substitutions per site. The analysis involved 48 nucleotide sequences. All positions containing gaps and missing data were eliminated. There were a total of 209 positions in the final dataset. Evolutionary analyses were conducted in MEGA6.
(TIF)

S5 Fig Consensus tree of Maximum Parsimony analysis of intron of *Aedes aegypti* (indel was treated as 5th variable). Evolutionary history was inferred using the Maximum Parsimony method using PHYLIP version 3.69. The consensus tree is shown. The percentages of replicate trees in which the associated sequences clustered together in the bootstrap test (1000 replicates) are shown next to the branches [57]. Branches corresponding to partitions reproduced in less than 50% trees (reproduced in only one tree) are collapsed.
(TIF)
