# Supplementary material for: Discovery of Point Mutations in the Voltage-Gated Sodium Channel from African Aedes aegypti Populations: Potential Phylogenetic Reasons for Gene Introgression
Source: PLoS Negl Trop Dis. 2016 Jun 15;10(6):e0004780. doi: 10.1371/journal.pntd.0004780 (PMC4909257; doi:10.1371/journal.pntd.0004780)
Supplement: S1 Table — (DOC) [file pntd.0004780.s001.doc]

**S1 Table. Information for the sequences between exon 20 and 21 of *Ae. aegypti*** used for phylogenetic analysis.

| Country – Code No. | Region | Accession number |
| --- | --- | --- |
| Brazil FJ479609 | South America | FJ479609 |
| Brazil FJ479610 | South America | FJ479610 |
| Brazil FJ479611 | South America | FJ479611 |
| Brazil FJ479613 | South America | FJ479613 |
| Brazil JX275501 | South America | JX275501 |
| El Salvador-1R^1)^ | Central America | LC036550 |
| Guatemala 1^1)^ | Central America | LC050222 |
| Ghana 001^1)^ | West Africa | LC036551 |
| Ghana 257^1)^ | West Africa | LC036552 |
| Kenya 2537 1-2^1)^ | East Africa | LC085630 |
| Kenya 603 2-4^1)^ | East Africa | LC085631 |
| Kenya 817 1-1^1)^ | East Africa | LC085632 |
| Kenya L040-2^1)^ | East Africa | LC085633 |
| Kenya L041-2^1)^ | East Africa | LC085634 |
| Kenya LU 7-1^1)^ | East Africa | LC085635 |
| Kenya Mbita 4-9^1)^ | East Africa | LC085636 |
| Kenya Mbita 5-6^1)^ | East Africa | LC085637 |
| Malawi 157^1)^ | South Africa | LC050219 |
| Malawi 187^1)^ | South Africa | LC050220 |
| Malawi 489^1)^ | South Africa | LC050221 |
| Zambia 002^1)^ | South Africa | LC085638 |
| Zambia 047^1)^ | South Africa | LC085639 |
| Zambia 286^1)^ | South Africa | LC085640 |
| Zambia 356^1)^ | South Africa | LC085641 |
| Zimbabwe 023^1)^ | South Africa | LC085642 |
| Zimbabwe 042^1)^ | South Africa | LC085643 |
| India KM677321 | South Asia | KM677321 |
| India KM677334 | South Asia | KM677334 |
| Indonesia KJ957878 | South East Asia | KJ957878 |
| Indonesia KJ957879 | South East Asia | KJ957879 |
| Indonesia KJ957881 | South East Asia | KJ957881 |
| Indonesia KJ957883 | South East Asia | KJ957883 |
| Myanmar AB914689 | South East Asia | AB914689 |
| Myanmar AB914690 | South East Asia | AB914690 |
| Philippines 004^1)^ | South East Asia | LC060441 |
| Philippines 005^1)^ | South East Asia | LC060442 |
| Philippines 015^1)^ | South East Asia | LC060443 |
| Philippines 020^1)^ | South East Asia | LC060444 |
| Philippines 056^1)^ | South East Asia | LC060445 |
| Philippines 379^1)^ | South East Asia | LC060446 |
| Philippines 435^1)^ | South East Asia | LC060447 |
| Singapore 01R^1)^ | South East Asia | LC036553 |
| Vietnam 1035-37-38-64-F01^1)^ | South East Asia | LC036554 |
| Vietnam 1087-73-66-68-R01^1)^ | South East Asia | LC036555 |
| Vietnam 1130-07-16R^1)^ | South East Asia | LC036556 |
| Vietnam 4166-32-05-55-48R^1)^ | South East Asia | LC036557 |
| Vietnam 4177-81-R02^1)^ | South East Asia | LC036558 |
| Vietnam 5030-35-R01^1)^ | South East Asia | LC036559 |

^1)^ Newly registered to GenBank in the present report.
