# Supplementary figures and images for: Discovery of Point Mutations in the Voltage-Gated Sodium Channel from African Aedes aegypti Populations: Potential Phylogenetic Reasons for Gene Introgression
Source: PLoS Negl Trop Dis. 2016 Jun 15;10(6):e0004780. doi: 10.1371/journal.pntd.0004780 (PMC4909257; doi:10.1371/journal.pntd.0004780)

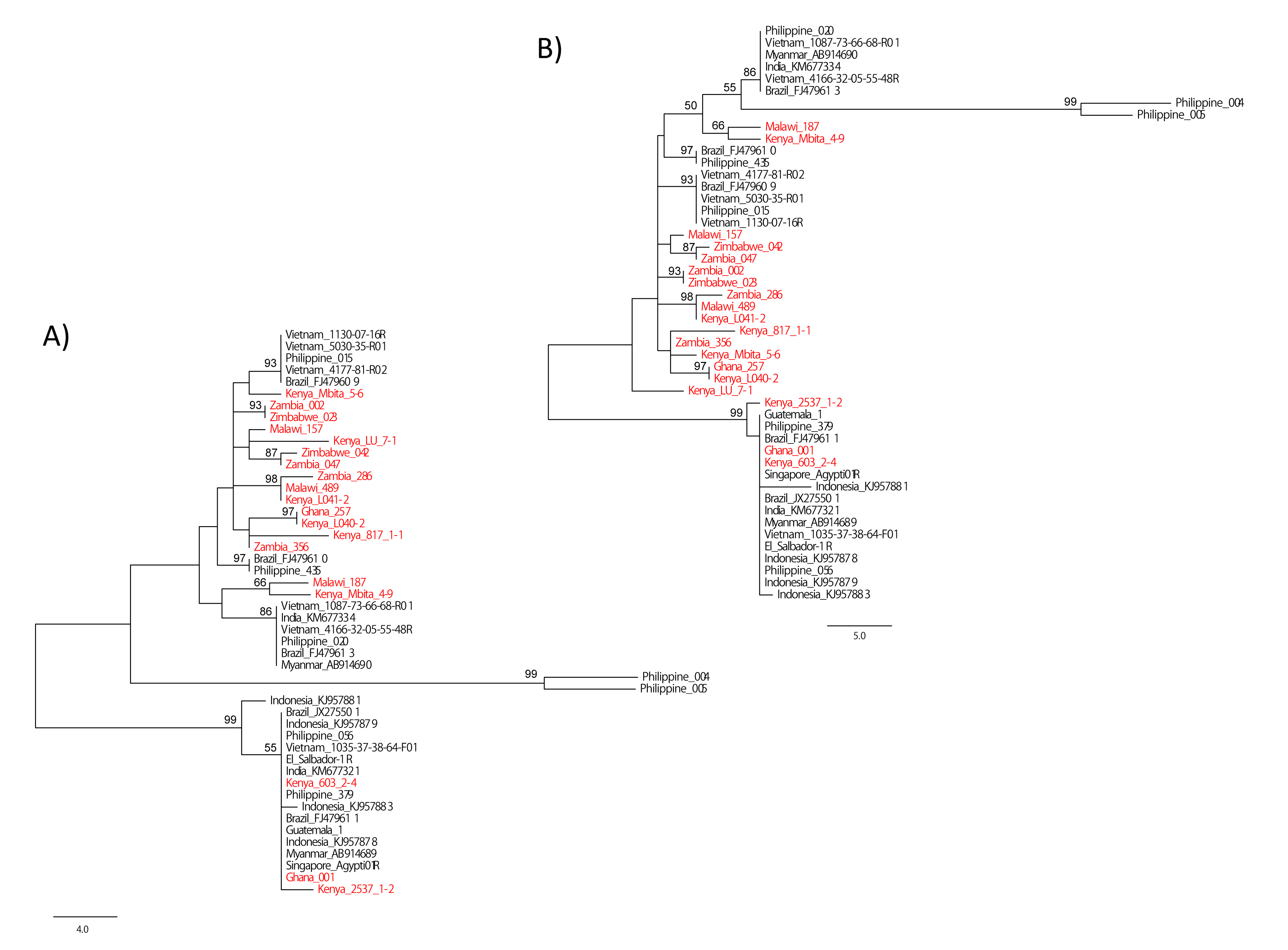

Supplement: S1 Fig — Other trees inferred using the Maximum Parsimony method are shown. Because of differences in two of them, we show only two trees. A) MP tree in which Philippine clade was placed at the base of clade 2, B) MP tree in which Philippine clade was NOT placed at the base of clade 2. The percentage of replicate trees in which the associated sequences clustered together in the bootstrap test (1,000 replicates) are shown next to the branches [56]. (TIF) [file pntd.0004780.s002.tif]

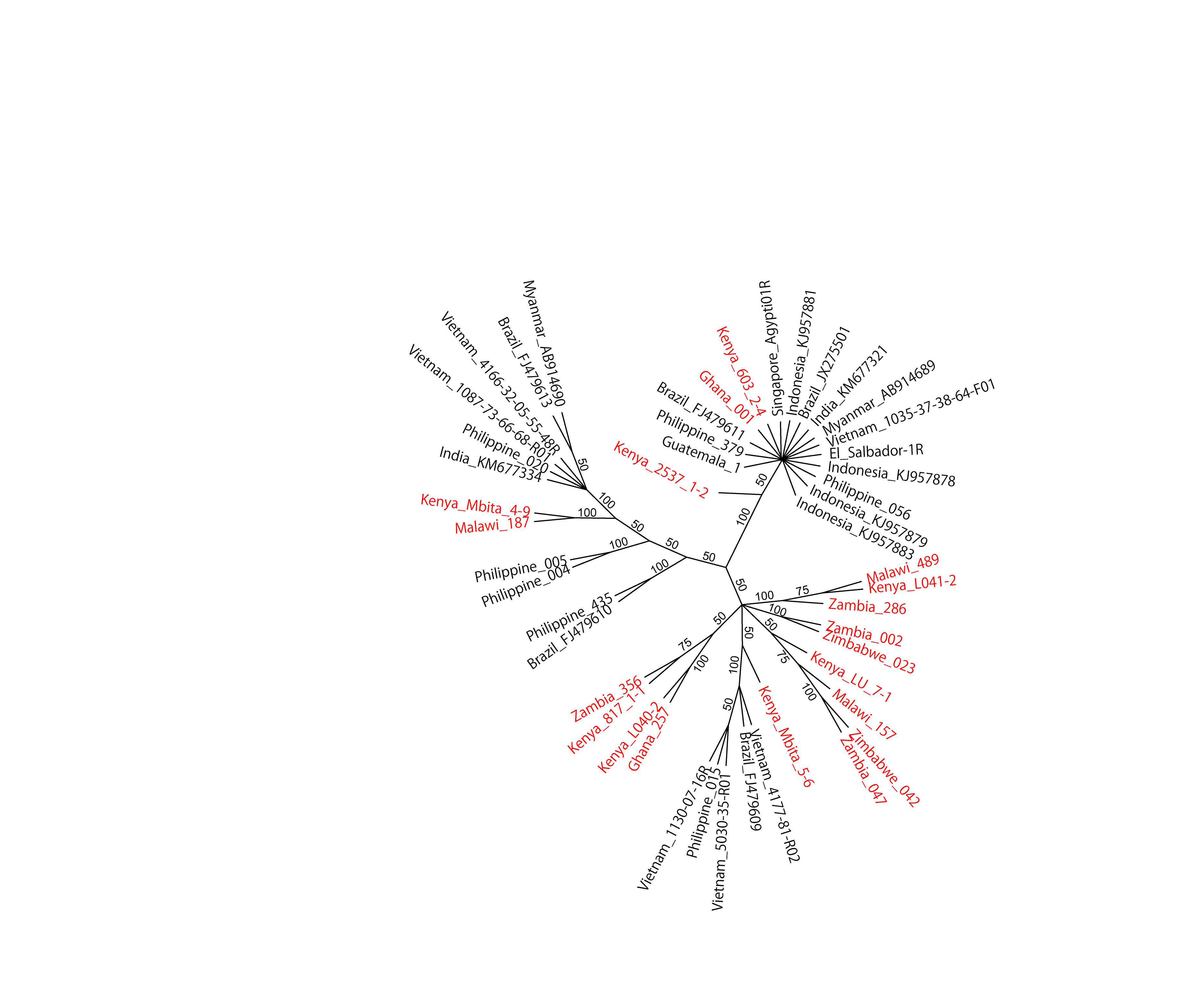

Supplement: S2 Fig — Evolutionary history was inferred using the Maximum Parsimony method. The consensus tree inferred from the four most parsimonious trees constructed using MEGA6 is shown. Branches corresponding to partitions reproduced in less than 50% trees (reproduced in only one tree) are collapsed. The percentages of parsimonious trees in which the associated sequences clustered together are shown next to the branches. (TIF) [file pntd.0004780.s003.tif]

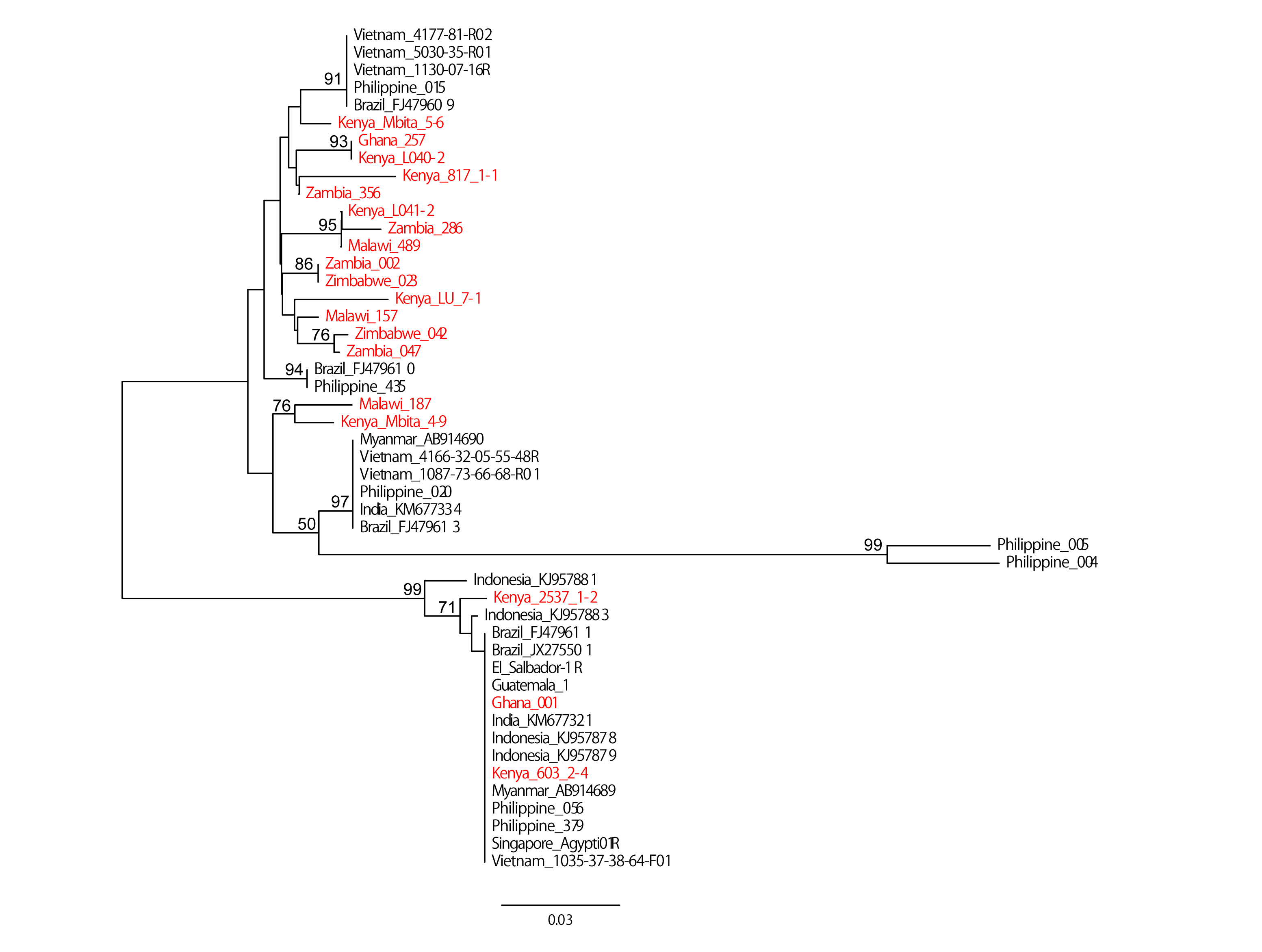

Supplement: S3 Fig — Evolutionary history was inferred using the Neighbor-Joining method [57]. The optimal tree with the sum of branch length = 0.566 is shown. The percentages of replicate trees in which the associated sequences clustered together in the bootstrap test (1,000 replicates) are shown next to the branches [54]. The tree is drawn to scale, with branch lengths in the same units as those of the evolutionary distances used to infer the phylogenetic tree. The evolutionary distances were computed using the Tamura 3-parameter method [58] and are in the units of the number of base substitutions per site. The analysis involved 48 nucleotide sequences. All positions containing gaps and missing data were eliminated. There were a total of 209 positions in the final dataset. Evolutionary analyses were conducted in MEGA6. (TIF) [file pntd.0004780.s004.tif]

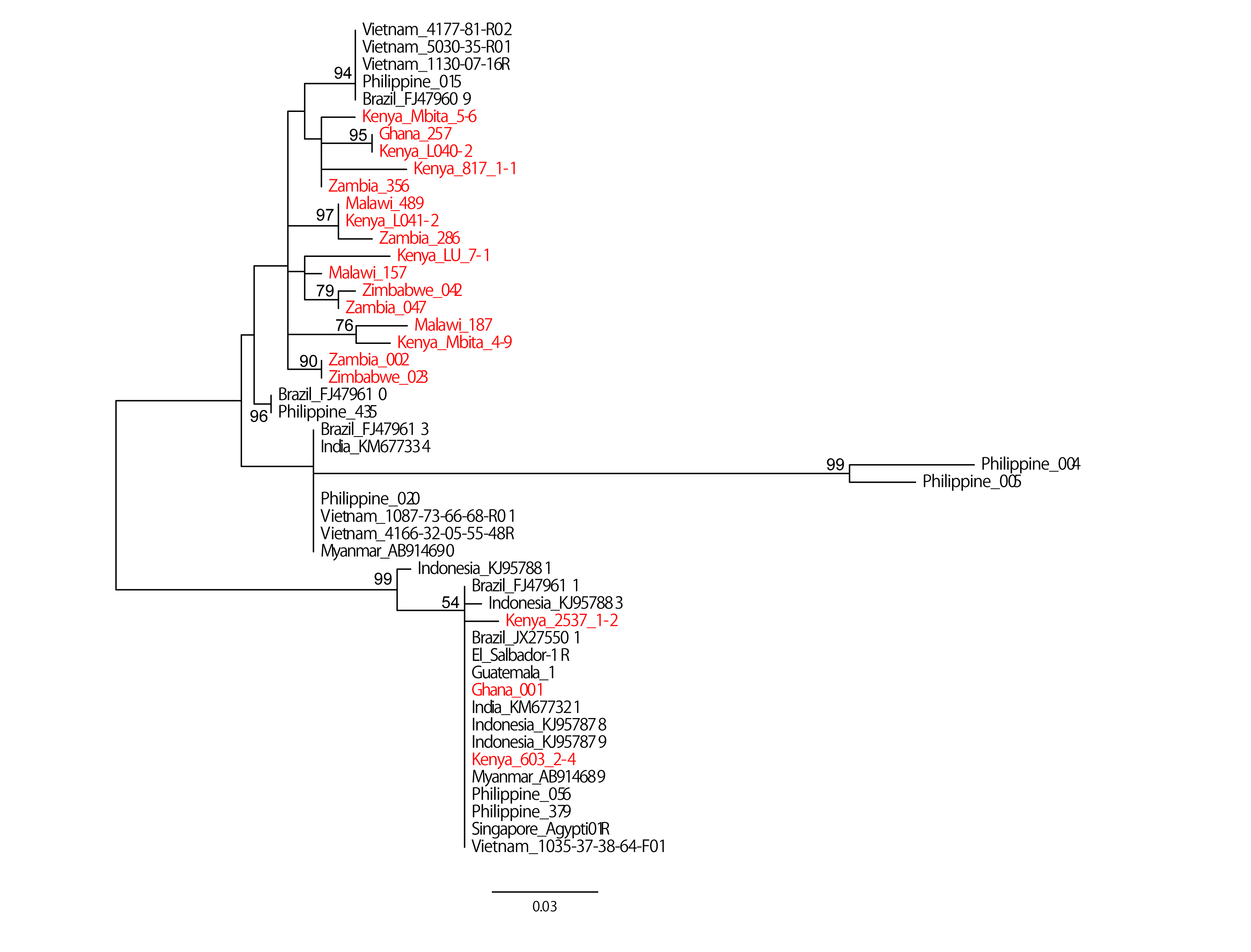

Supplement: S4 Fig — Evolutionary history was inferred by using the Maximum Likelihood method based on the Tamura 3-parameter model [58]. The tree with the highest log likelihood (-899.5569) is shown. The percentages of replicate trees in which the associated sequences clustered together in the bootstrap test (1,000 replicates) are shown next to the branches [56]. Initial tree(s) for the heuristic search were obtained by applying the Neighbor-Joining method to a matrix of pairwise distances estimated using the Maximum Composite Likelihood (MCL) approach. The tree is drawn to scale, with branch lengths measured in the number of substitutions per site. The analysis involved 48 nucleotide sequences. All positions containing gaps and missing data were eliminated. There were a total of 209 positions in the final dataset. Evolutionary analyses were conducted in MEGA6. (TIF) [file pntd.0004780.s005.tif]

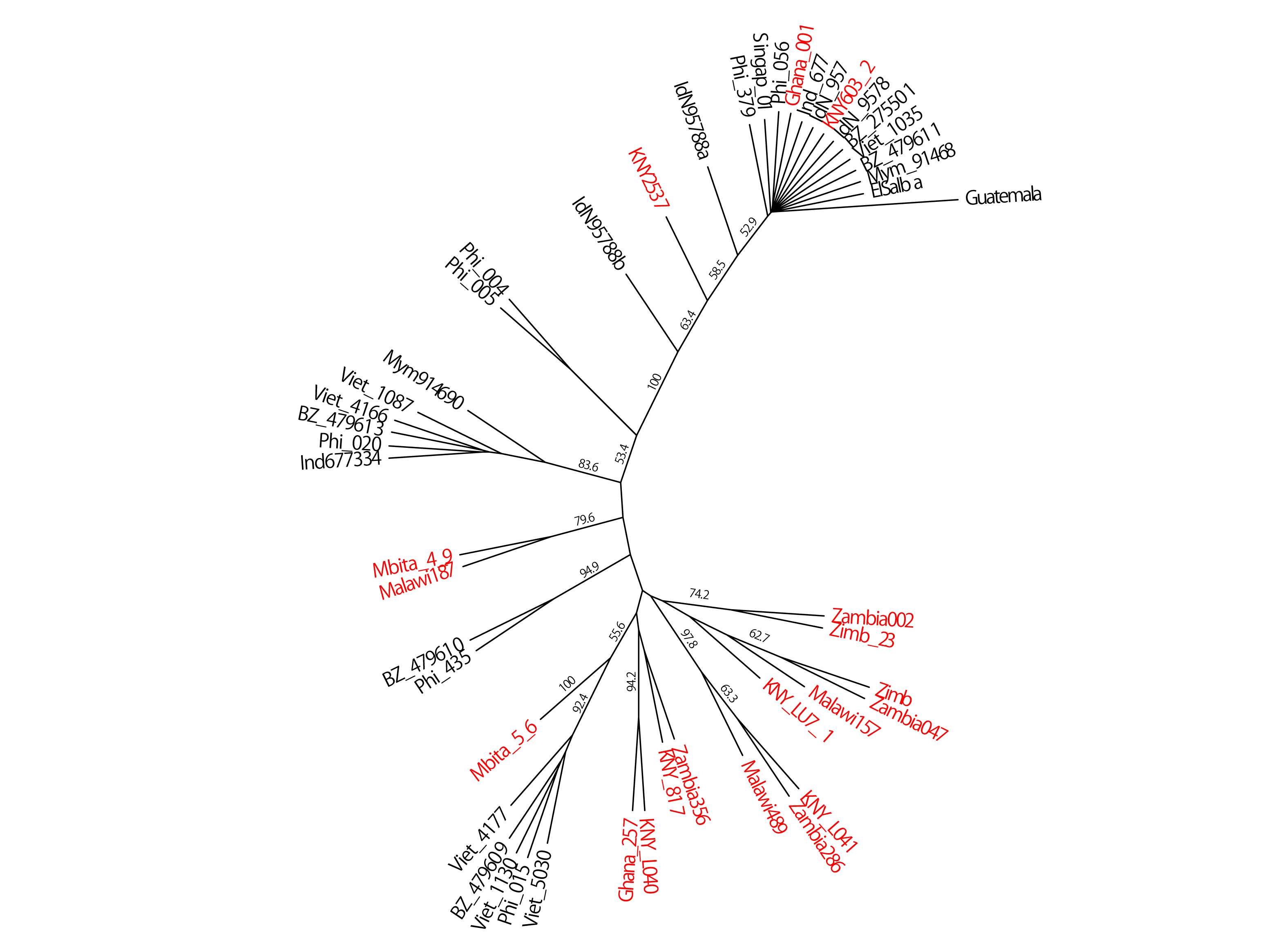

Supplement: S5 Fig — Evolutionary history was inferred using the Maximum Parsimony method using PHYLIP version 3.69. The consensus tree is shown. The percentages of replicate trees in which the associated sequences clustered together in the bootstrap test (1000 replicates) are shown next to the branches [57]. Branches corresponding to partitions reproduced in less than 50% trees (reproduced in only one tree) are collapsed. (TIF) [file pntd.0004780.s006.tif]
